# Serum Clusterin: A Potential Marker for Assessing the Clinical Severity and Short-Term Prognosis of Hepatitis B Virus-Related Acute-on-Chronic Liver Failure

**DOI:** 10.1155/2020/8814841

**Published:** 2020-12-12

**Authors:** Huimin Liu, Yuxin Li, Fangyuan Gao, Peipei Meng, Hao Yu, Tong Wu, Yang Zhou, Yuyong Jiang, Xianbo Wang

**Affiliations:** Center of Integrative Medicine, Beijing Ditan Hospital Affiliated to Capital Medical University, Beijing 100015, China

## Abstract

**Background:**

Acute-on-chronic liver failure (ACLF) is a clinical syndrome characterized by acute deterioration of liver function and high short-term mortality. Clusterin, with biological functions similar to small heat shock proteins, can protect cells from apoptosis induced by various stressors. The aim of this study was to detect the level of serum clusterin in hepatitis B virus- (HBV-) related ACLF and to assess the predictive value of clusterin for the short-term prognosis of HBV-ACLF.

**Methods:**

We detected serum clusterin by ELISA in 108 HBV-ACLF patients, 63 HBV-non-ACLF patients, and 44 normal controls.

**Results:**

Serum clusterin was markedly lower in HBV-ACLF patients (median, 51.09 *μ*g/mL) than in HBV-non-ACLF patients (median, 188.56 *μ*g/mL) and normal controls (median, 213.45 *μ*g/mL; all *P* < 0.05). Nonsurviving HBV-ACLF patients who died within 90 days had much lower clusterin levels than did surviving patients, especially those who died within 28 days (nonsurvival group vs. survival group: 39.82 ± 19.34 vs. 72.26 ± 43.52, *P* < 0.001; survival time ≤ 28 vs. survival time > 28: median 28.39 vs. 43.22, *P* = 0.013). The results showed that for identifying HBV-ACLF, the sensitivity of clusterin (93.7%) was similar to the sensitivities of the international normalized ratio (INR; 94.4%) and total bilirubin (TBIL; 94.8%), but its specificity (90.7%) was higher than that of prothrombin activity (PTA; 65.8%) and TBIL (69.8%) and was similar to INR (88.9%). As the concentration of clusterin increased, the mortality of HBV-ACLF patients decreased significantly from 59.3% to 7.0%. Clusterin had better ability for predicting the prognosis of HBV-ACLF patients than did the model for end-stage liver disease (MELD) score and the chronic liver failure consortium (CLIF-C) ACLF score (MELD vs. clusterin: *P* = 0.012; CLIF-C ACLF vs. clusterin: *P* = 0.031).

**Conclusion:**

Serum clusterin is a potential biomarker for HBV-ACLF which can be used to assess clinical severity and the short-term prognosis of patients with this disease and may help clinicians identify HBV-ACLF with greater specificity and improved prognostic accuracy than existing prognostic markers.

## 1. Introduction

Acute-on-chronic liver failure (ACLF) is a clinical syndrome characterized by acute deterioration of liver function, multiple organ failure (liver, brain, and kidney, caused by coagulation, circulation, and/or respiration dysfunction), and a high short-term mortality rate (>15% at 28 days) [1, 2], which seriously threatens the life and health of patients with chronic liver diseases. ACLF frequently occurs in alcoholic cirrhosis in Europe and North America, whereas hepatitis B virus- (HBV-) related ACLF (HBV-ACLF) is the main type found in the Asia-Pacific and African regions [1, 3]. The European chronic liver failure consortium (CLIF) CANONIC study of ACLF in cirrhosis showed that systemic inflammation is remarkable in ACLF. Systemic inflammation induces ACLF through complex mechanisms, such as excessive inflammatory response and systemic oxidative stress to pathogen-associated molecular patterns, which is typical pathogenesis in patients who have ACLF derived from alcoholic liver cirrhosis and which correlates with multiple organ failure and mortality [1, 4]. The critical histological features of HBV-related ACLF have been identified as submassive hepatic necrosis and systemic inflammatory responses induced by necrotic hepatocytes and bacteria. These features also promote the progression of the disease and organ failure, with a mechanism similar to alcoholic ACLF [1, 4]. At present, there are still no unified diagnostic criteria and treatment programs for different etiologies of ACLF. Current management and therapeutic programs for ACLF are composed of treatment for associated complications, organ failure support, and liver transplantation.

Clusterin, also named apolipoprotein J or sulfated glycoprotein-2, is a 75–80 kDa disulfide-linked heterodimeric glycoprotein widely expressed in various tissues and body fluids [5, 6]. It has two isoforms: a secretory extracellular form and a precursor nuclear form. The secretory clusterin is abundant in biological fluids such as seminal fluid, plasma, milk, and cerebrospinal fluid. Secretory clusterin has many biological functions similar to small heat shock proteins and is involved in a number of biological processes such as lipid transport, tissue remodeling, cell apoptosis, and reproduction [7, 8]. It has been reported that clusterin plays an important role in attenuating hepatic fibrosis by inhibiting the activation of hepatic stellate cells and Smad3 signaling pathways [9]. Clusterin is also overexpressed in many cancers, thereby protecting cells from apoptosis induced by chemotherapy or radiotherapy, and furthermore, clusterin influences the invasion, metastasis, and transdifferentiation of tumors [10–13]. Recently, some studies have shown that inhibiting clusterin can enhance sensitivity to chemotherapy [14, 15]. Clusterin also plays a significant role in inflammation and immune responses through molecular interactions [16, 17].

In a previous proteomic analysis of HBV-ACLF plasma, we found that the clusterin level was lower in HBV-ACLF patients compared with chronic HBV patients [18]. Based on the multiple biological functions of clusterin and the pathological features of ACLF, we hypothesize that serum clusterin may play an important role in the pathogenesis of ACLF and may serve as a novel biomarker for acute-on-chronic liver failure. In the current study, we detected the serum level of clusterin in HBV-ACLF and assessed the predictive value of clusterin for the short-term prognosis of HBV-ACLF.

## 2. Material and Methods

### 2.1. Study Subjects

From January 1, 2018, to June 30, 2019, we prospectively collected peripheral blood serum samples from patients with chronic HBV infection who were admitted to Beijing Ditan Hospital Affiliated to Capital Medical University (Beijing, China) for the first time after appearing with severe abnormal liver function, including 108 patients with HBV-ACLF and 63 patients with HBV-non-ACLF. Samples were also collected from 44 healthy volunteers (normal controls), who were verified as disease-free by physical examination. The ethics committee of Beijing Ditan Hospital approved the study protocol according to the Declaration of Helsinki and supervised the implementation of the project.

### 2.2. Diagnostic Criteria

The definition of acute-on-chronic liver failure proposed by the Asian Pacific Association for the Study of the Liver (APASL) is that ACLF is an acute hepatic insult manifesting as jaundice (serum bilirubin ≥ 5 mg/dL (85 *μ*mol/L)) and coagulopathy (international normalized ratio (INR) ≥ 1.5 or prothrombin activity (PTA) < 40%) complicated within 4 weeks by clinical ascites and/or encephalopathy in a patient with previously diagnosed or undiagnosed chronic liver disease/cirrhosis and is associated with a high 28-day mortality [3]. HBV-ACLF refers to ACLF caused by activation of HBV. HBV-non-ACLF refers to patients with chronic hepatitis B (CHB), reactivation of HBV, or HBV-related liver cirrhosis, who have abnormal liver function due to chronic HBV infection, but do not meet the diagnostic criteria of ACLF according to APASL. CHB was defined as chronic necroinflammatory disease of the liver caused by persistent infection with HBV for more than six months, which can be subdivided into HBeAg-positive and HBeAg-negative CHB [19]. The reactivation of HBV was diagnosed by an acute increase in HBV-DNA and aminotransferase in patients on continuous treatment with nucleotide analogs (NUCs) following cessation or resistance [20]. The reactivation of HBV can be spontaneous or triggered by intensive chemotherapy, hormones, or immunosuppressive drugs [3]. Cirrhosis was diagnosed based on clinical symptoms, laboratory tests, and CT/MRI scan or liver biopsy [21].

### 2.3. Inclusion and Exclusion Criteria

Inclusion criteria include the following: (1) patients presenting with HBV-ACLF or HBV-non-ACLF with chronic HBV infection, including patients with CHB, or reactivation of HBV, or HBV-related liver cirrhosis; (2) patients who first came to Beijing Ditan Hospital when the abnormal liver function occurred and had not been treated in other hospitals; (3) subjects aged 18–60 years old; and (4) HBV-non-ACLF patients who had alanine aminotransferase (ALT) or aspartate aminotransferase (AST) levels > 2 times the upper limit of normal (40 U/L) or a total bilirubin (TBIL) level > 2 times the upper limit of normal (1.1 mg/dL).

Exclusion criteria include the following: (1) hepatitis A, C, D, or E virus coinfection; (2) liver injury with no HBV infection, including patients with liver damage or failure caused by alcohol, hepatotoxic drugs, steatohepatitis, infection or autoimmune diseases, and other causes; (3) other diseases (respiratory diseases, cardiovascular and cerebrovascular diseases, kidney disease, autoimmune diseases, and malignant tumors) that could affect outcomes; (4) primary or secondary liver cancer; (5) patients who prepared to undergo transplantation within 48 h after enrollment; and (6) pregnant women and those taking any other medication not related to the disease at the time of the study.

The healthy volunteers were 18–60 years old, and they were considered healthy as judged by a physician on the basis of the medical history, laboratory testing, and physical examination.

### 2.4. Treatment

All HBV-ACLF patients received standard treatments, including antiviral therapy by nucleos(t)ide analogs, intravenous infusions of albumin, their own blood type of plasma, and nutrition support [3]. Patients in the HBV-non-ACLF group received conventional liver protection treatment and antiviral therapy [19, 21]. Entecavir or tenofovir was preferred for the HBV-ACLF and HBV-non-ACLF patients without prior antiviral therapy and also for those who discontinued antiviral drugs. Patients with HBV resistance were treated with combination NUCs, or the NUCs were replaced by other nucleos(t)ide analogs according to their previous medication.

The therapy for associated complications of ACLF or decompensated cirrhosis, including abdominal infection, hepatic encephalopathy (HE), unbalanced electrolytes, and hepatorenal syndrome, was also carried out according to relevant guidelines [3]. Artificial liver therapy was available if medical treatment was not effective.

### 2.5. Data Collection

Baseline clinical information and laboratory variables were collected from subjects after ACLF or non-ACLF was diagnosed, including sex, age, survival serum alanine transaminase, aspartate transaminase, TBIL, glutamyl transpeptidase, white blood cell count (WBC), neutrophil count (NC), lymphocyte count (LC), PTA, INR, hepatitis B e antigen, HBV DNA, and serum creatinine (Cr). Complications, including spontaneous bacterial peritonitis (SBP), hyponatremia, HE, and hepatorenal syndrome (HRS), were also recorded. The model for end-stage liver disease (MELD) score and the chronic liver failure consortium ACLF (CLIF-C ACLF) score were calculated from the laboratory data [22, 23]. Laboratory tests with missing data were not included in the analysis.

Based on the survival outcome at 28 days and 90 days after hospitalization, the patients in each time point were further divided into survival and nonsurvival groups. All patients were monitored after diagnosis either until their death or the end of the 90-day follow-up period. All survival patients at 90 days were followed for 1 year.

### 2.6. Measurements of Serum Clusterin

Serum samples from all patients were collected on the first day after diagnosis. The venous blood samples were drawn with a serum separator tube from the fasting patients in the morning from 6:30 AM to 7:30 AM, allowed to clot for 30 min at room temperature, and then centrifuged (1000 g, 4°C, 15 min) in the laboratory. Blood sera were aspirated and stored at −80°C. Serum concentration of secretory clusterin was measured with the Human Clusterin Quantikine ELISA Kit (DCLU00, R&D, USA) following the manufacturer protocol. Three replicates from each sample were tested, and the average was calculated.

### 2.7. Statistical Analysis

SPSS 18.0 (SPSS, IBM, USA) software was used to perform statistical analysis, and all statistical tests were two-sided. *P* < 0.05 was considered statistically significant. Categorical variables were reported as frequency and percentage in each group. Normally distributed variables were represented as means ± standard deviations (SD), and nonnormally distributed variables were represented as medians with interquartile ranges. Independent sample *t*-tests, Chi-squared tests, nonparametric tests, or one-way analysis of variance were used for comparing differences among groups, depending on the type of variable. All variables showing clinical and statistical significance were selected for multivariate analysis. A logistic regression model, using the forward-LR method, was applied to screen for independent influencing factors associated with the outcome. Test accuracy was assessed by the area under the receiver operating characteristic (ROC) curve (AUC).

## 3. Results

### 3.1. Characteristics of Study Subjects

A total of 215 patients and healthy participants were recruited in our study and were classified as HBV-ACLF (*n* = 108), HBV-non-ACLF (*n* = 63), and normal controls (*n* = 44). In the HBV-ACLF group, there were 85 males and 23 females, age range 21–59 years, with mean age of 43.91 ± 10.37 years. In the HBV-non-ACLF group, there were 52 males and 11 females, age range 20–60 years, with mean age of 38.42 ± 12.67 years. The mean age of 35 males and nine females in the normal control group was 39.16 ± 13.11, age range 21–60. Characteristics of HBV-ACLF and HBV-non-ACLF patients and normal controls are shown in Table 1. There were no significant differences in the mean age, gender ratio, and serum Cr among the three groups (*P* > 0.05, Table 1). Comparing HBV-non-ACLF patients with normal controls, the patients had the highest values for ALT, AST, TBIL, WBC, NC, INR, MELD score, and neutrophil-lymphocyte ratio (NLR; *P* < 0.05, Table 1). There was a significant reduction in albumin, LC, PTA, and clusterin (*P* < 0.05, Table 1) in HBV-ACLF patients compared with HBV-non-ACLF patients and normal controls (*P* < 0.05, Table 1). No patients with NUC resistance or HBV reactivation induced by hormone or immunosuppressive drugs were enrolled in this study. Thus, both the non-ACLF and ACLF groups were composed of patients with HBV spontaneous clearance and NUC cessation, and there was no statistical difference in the constituent ratios between the two groups (*P* > 0.05, Table 1).

**Figure 1:**
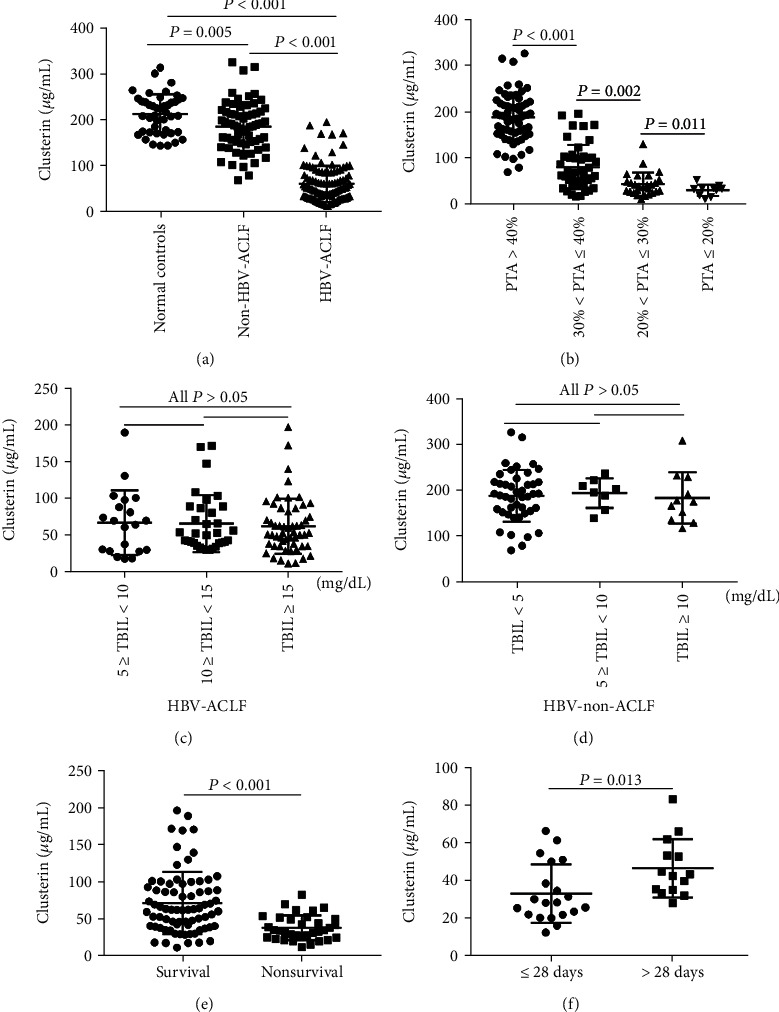
Comparisons of serum clusterin levels among different groups. (a) The levels of serum clusterin in HBV-ACLF and HBV-non-ACLF patients and normal controls. (b) The levels of serum clusterin in HBV-ACLF patients with different levels of PTA. (c) The levels of serum clusterin in HBV-ACLF patients with different levels of TBIL. (d) The levels of serum clusterin in HBV-non-ACLF patients with different levels of TBIL. (e) The levels of serum clusterin in HBV-ACLF survival and nonsurvival groups. (f) The levels of serum clusterin in HBV-ACLF patients with survival time ≤ 28 days and >28 days. Abbreviations: HBV-ACLF: hepatitis B virus-related acute-on-chronic liver failure; PTA: prothrombin activity; TBIL: total bilirubin.

There were significant differences in clusterin, PTA, WBC, NC, LC, NLR, INR, TBIL, and incidences of HE and hepatorenal syndrome between HBV-non-ACLF and HBV-ACLF patients (*P* < 0.05, Table 1). The number of HBV e antigen-positive patients and the levels of HBV DNA were not significantly different between the HBV-ACLF and HBV-non-ACLF groups (*P* > 0.05, Table 1). In particular, among the three groups, the level of clusterin was highest in normal controls. Patients with HBV-non-ACLF also displayed higher expression of clusterin than those with HBV-ACLF (normal controls vs. HBV-non-ACLF: 213.45 *μ*g/mL (range 144.57–313.48) vs. 188.56 *μ*g/mL (70.22–325.36), *P* = 0.005; HBV-ACLF vs. HBV-non-ACLF: 51.09 *μ*g/mL (12.17–188.62)) vs. 188.56 *μ*g/mL (70.22–325.36), *P* < 0.001 (Figure 1(a))).

### 3.2. Association between Serum Clusterin Level and Clinical Pathologic Parameters in HBV-ACLF

We analyzed the association between serum clusterin level and multiple clinical pathologic parameters. We found that the expression level of clusterin was not significantly different between the following groups: individuals above and below 55 years of age (Table 2), male and female (Table 2), serum Cr above and below 1.5 mg/dL (Table 2), and pathological basis of chronic hepatitis and liver cirrhosis (Table 2). There were also no significant differences in the level of clusterin between HBV-ACLF patients with and without bacterial infection (Table 2) and HBV DNA above and below 10 log_10_ IU/mL (Table 2).

All HBV-ACLF patients were divided into three groups according to the value of PTA and were then compared with HBV-non-ACLF patients (PTA > 40%). The expression level of clusterin in HBV-ACLF patients with 30% < PTA ≤ 40% was higher than those with 20% < PTA ≤ 30% (*P* = 0.002, Table 2 and Figure 1(b)) and higher than those with PTA ≤ 20% (*P* < 0.001, Table 2 and Figure 1(b)). The clusterin level of HBV-ACLF patients with 20% < PTA ≤ 30% was also higher than those with PTA ≤ 20% (*P* = 0.011, Table 2 and Figure 1(b)). We also compared serum clusterin in HBV-ACLF and HBV-non-ACLF patients who had different levels of serum TBIL. There were no statistically significant differences in serum clusterin concentrations between patients with HBV-ACLF (Table 2) or HBV-non-ACLF with different levels of TBIL (all *P* > 0.05, Figures 1(c) and 1(d)).

### 3.3. Association of Serum Clusterin with Outcomes of HBV-ACLF

**Figure 2:**
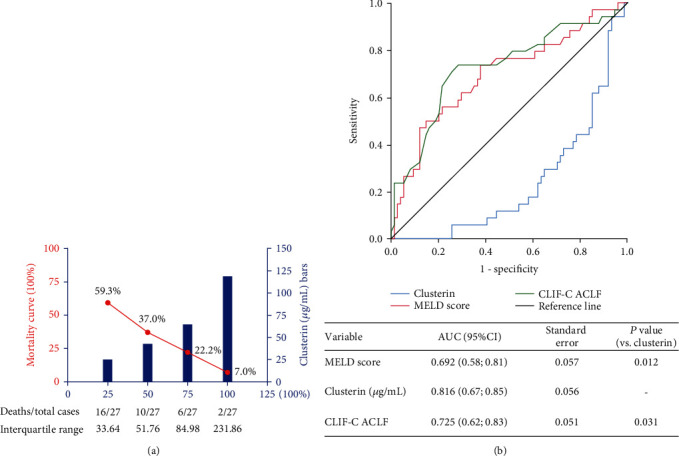
The correlation between serum clusterin levels and mortality of patients with HBV-related acute-on-chronic liver failure and value of serum clusterin for predicting short-term prognosis of hepatitis B virus-related acute-on-chronic liver failure. (a) Comparison of mortality in HBV-ACLF patients between four different concentration gradients of serum clusterin. (b) The AUCs of MELD score, CLIF-C ACLF score, and clusterin for predicting prognosis of HBV-ACLF. Abbreviations: HBV-ACLF: hepatitis B virus-related acute-on-chronic liver failure; MELD: model for end-stage liver disease; CLIF-C ACLF: chronic liver failure consortium on acute chronic liver failure.

To further understand the significance of clusterin expression in HBV-ACLF, we explored the association between levels of clusterin and the prognosis for HBV-ACLF patients. Based on the outcomes at the 90-day follow-up, the HBV-ACLF patients were divided into survival and nonsurvival groups. The analysis showed that the 90-day nonsurvival group had a low clusterin level compared with the survival group (nonsurvival group vs. survival group: 39.82 ± 19.34 *μ*g/mL vs. 72.26 ± 43.52 *μ*g/mL, *P* < 0.001, Table 2 and Figure 1(e)). We further explored the relationship between the expression level of serum clusterin and survival time. The HBV-ACLF patients with survival times of more than 28 days after diagnosis had significantly higher clusterin levels compared with patients who died within 28 days (survival time ≤ 28 days vs. survival time > 28 days: median 28.39 *μ*g/mL vs. 43.22 *μ*g/mL, *P* = 0.013, Table 2 and Figure 1(f)). We then divided clusterin levels of the HBV-ACLF patients into four concentration gradients based on the interquartile range and compared the mortalities of the different clusterin concentration groups. As serum clusterin concentration increased, the mortality of patients with HBV-ACLF decreased significantly from 59.3% to 7.0% (Figure 2(a)).

### 3.4. Serum Clusterin Is a Potential Biomarker for HBV-ACLF

Our data showed that the level of serum clusterin in HBV-ACLF patients was lower than that in HBV-non-ACLF patients and normal controls. To further reveal the implications of clusterin expression in HBV-ACLF, multivariate analysis of variables was performed by placing into the logistic regression all clinical variables comparing HBV-non-ACLF and HBV-ACLF patients with *P* less than 0.05 in Table 1, including clusterin, HE, PTA, WBC, NC, LC, NLR, INR, and TBIL. Only clusterin, PTA, and TBIL were independent influencing factors for HBV-ACLF (Table 3). To evaluate the efficiency of clusterin for identifying HBV-ACLF, we compared the areas under the ROC curve, sensitivity, and specificity.  Table 4 illustrates the sensitivities and specificities of single factors utilizing INR (≥1.5), PTA (≤40%), or TBIL (≥5 mg/dL) for determining HBV-ACLF (sensitivities: INR, 94.4%; PTA, 98.4%; and TBIL, 94.8%; specificities: INR, 88.9%; PTA, 65.8%; and TBIL, 69.8%). The sensitivities and specificities of clusterin with a cutoff value of 105 *μ*g/mL for determining HBV-ACLF were 93.7% and 90.7%, respectively. All the recognized parameters as well as clusterin had large AUCs (Table 4, INR: 0.976; PTA, 0.975; TBIL, 0.888; and clusterin, 0.964), which indicates that they were accurate for identifying ACLF.

### 3.5. Predictive Value of Serum Clusterin for Prognosis of HBV-ACLF

The significant difference in serum clusterin between nonsurvival and survival groups of patients suggested that predicting the prognosis of HBV-ACLF by clusterin was possible and meaningful. In univariate analysis, the 90-day mortality rate was associated with several clinical variables, including age, TBIL, PTA, INR, NLR, MELD score, clusterin, HE, and HRS (Table 5). In multivariate analysis, independent predictive factors of a poor outcome in HBV-ACLF were age, clusterin, HE, and TBIL (Table 6). The ROC curve was used to evaluate the predictive value of clusterin for the prognosis of HBV-ACLF (Figure 2(b)). The data demonstrated that the AUCs of clusterin, MELD score, and CLIF-C ACLF score were 0.816 (95% confidence intervals (CI): 0.67–0.85), 0.692 (95% CI: 0.58–0.81), and 0.725 (95% CI: 0.62–0.83), respectively. Clusterin had a larger AUC compared with the MELD score and CLIF-C ACLF score (Figure 2(b), MELD vs. clusterin: *P* = 0.012; CLIF-C ACLF vs. clusterin: *P* = 0.031).

## 4. Discussion

This study primarily focused on serum clusterin levels in patients with HBV-ACLF and showed that clusterin was closely associated with the prognosis of HBV-ACLF. The main finding of our study was that the serum clusterin level was significantly lower in HBV-ACLF patients, especially in those who died within 90 days or had survival times less than 28 days after diagnosis. Serum clusterin may thus be useful as a new biomarker for estimating clinical severity and 90-day adverse prognosis in HBV-ACLF. Furthermore, we found that clusterin may help clinicians identify HBV-ACLF patients with greater specificity. Our study also showed that age, clusterin, HE, and TBIL were independent predictive factors associated with 90-day mortality in HBV-ACLF patients. Compared with the MELD score and CLIF-C ACLF score, serum clusterin demonstrated better predictive accuracy.

Clusterin is secreted by human hepatocytes, which are the major source of circulating clusterin [24]. Serious liver damage directly leads to decreased synthesis of clusterin. Thus, the clusterin level in HBV-ACLF patients was significantly lower than that in HBV-non-ACLF patients, suggesting that the expression level of clusterin directly reflects the severity of liver injury. In addition, the results of multivariate analysis showed that clusterin, PTA, and TBIL were independent factors for identifying HBV-ACLF. Clusterin concentration may thus have implications for the clinical diagnosis of ACLF. We compared PTA, INR, TBIL, and clusterin by their AUCs. TBIL is one of the important laboratory indices in ACLF patients, which should be combined with PTA or INR for diagnosis of ACLF, according to the APASL consensus recommendation [3]. TBIL and PTA alone have high sensitivity, but their specificity is too low for identifying ACLF. We observed that the level of clusterin was strongly associated with PTA levels and increased with PTA in HBV-ACLF patients, but there was no significant association between serum clusterin and TBIL in HBV-ACLF patients or HBV-non-ACLF patients. The ROC showed a cutoff value for clusterin at 105 with high sensitivity (93.7%) and specificity (90.7%) for identifying ACLF. This suggests that when patients with acute exacerbations of chronic liver disease have high TBIL (>5 mg/dL), clusterin can help clinicians identify patients with ACLF with sensitivity similar to PTA but with higher specificity than PTA.

Hepatocyte apoptosis and necrosis mediated by many pathways are the key to the occurrence and development of hepatic failure. The occurrence of ACLF is often accompanied by complicated host immune dysregulation, in which abnormal adaptive and innate immune responses play an important role in mediating hepatic inflammation and hepatocyte apoptosis [25–27]. Clusterin can protect cells from apoptosis induced by various stressors [17]. In the mitochondrial apoptosis pathway, clusterin stabilizes the Ku70-Bax complex, preventing the apoptotic protein Bax from binding to the mitochondrial outer membrane, thereby blocking its proapoptotic activity [28]. Studies have shown that clusterin protects against cell death by reducing the cytotoxic effect of TNF-*α* in LNCap cells [29], and there was a relationship between the expressed level of clusterin and the degree of TNF-*α*-induced apoptosis [7, 30]. In the current study, clusterin levels in HBV-ACLF and HBV-non-ACLF patients were lower than in normal controls. With greater severity of liver injury, the expression levels of clusterin in HBV-ACLF patients were significantly lower than those in HBV-non-ACLF patients. We also found that HBV-ACLF patients who died within 90 days had much lower clusterin levels than did surviving patients, especially in those who died within 28 days. These results suggest that serum clusterin, as an inhibitor of apoptotic proteins, may stop the progression of disease by protecting hepatocytes against apoptosis and injury. The low expression of clusterin in HBV-ACLF patients could not effectively block activation of the apoptosis pathway, resulting in substantial hepatocellular apoptosis and necrosis. Therefore, HBV-ACLF patients with lower levels of clusterin tended to have more severe disease. The lower serum clusterin levels heralded shorter survival time within 90 days and more adverse short-term prognosis. Clusterin may thus be a potential therapeutic target for ACLF given its role in antiapoptotic processes.

Kidney failure is a component of the ACLF syndrome, and clusterin might be affected by renal function, but we did not find any significant difference between HBV-ACLF patients with normal or abnormal serum Cr. In this study, we compared the levels of clusterin expression in HBV-ACLF patients with serum Cr > 1.5 mg/dL and ≤1.5 mg/dL. There were no significant differences between these two groups. In addition, we did not find any difference in clusterin expression between HBV-ACLF patients with HBV DNA (log_10_ IU/mL ≤ 10) and those with HBV DNA > 10, which showed that clusterin expression was not directly affected by the HBV DNA level.

The following are some limitations of this study: Firstly, the study was based on the patients who were diagnosed by APASL ACLF diagnostic criteria, and all data were obtained from a single institution in China. Secondly, this study verified the results we reported in our previous proteomic analysis of HBV-ACLF plasma. Although clusterin is a potential indicator for identifying HBV-ACLF and predicting the prognosis of HBV-ACLF patients, we believe that a larger sample size should be examined because of the limited number of patients included in this study. Furthermore, the exact cutoff value of clusterin should also be obtained for clinical application.

## 5. Conclusion

In conclusion, our study indicates that clusterin is a potential biomarker for assessing clinical severity and short-term prognosis of patients with HBV-ACLF, which may help clinicians identify HBV-ACLF with higher specificity and improve the predictive accuracy of existing prognostic markers. Measurement of serum clusterin may be helpful in clinical practice for making treatment decisions for patients with HBV-ACLF.

## Figures and Tables

**Table 1:** Clinical baseline characteristics of study subjects.

Characteristics	Normal controls (*n* = 44)	HBV-non-ACLF (*n* = 63)	HBV-ACLF (*n* = 108)	*P* value
Age (years)	39.16 ± 13.11	38.42 ± 12.67	43.91 ± 10.37	0.157
Male, *n* (%)	35 (79.5%)	52 (82.53%)	85 (78.70%)	0.236
Alanine aminotransferase (U/L)	19.20 (15.00, 32.70)	451.60 (55.00, 1768.70)^∗∗∗^	502.15 (297.0, 1863.2)	<0.001
Aspartate aminotransferase (U/L)	18.70 (11.70, 35.10)	434.80 (76.20, 1714.90)^∗∗∗^	452.40 (334.0, 1520.7)	<0.001
Total bilirubin (*μ*mol/L)	12.25 (3.70, 15.50)	89.23 ± 53.29^∗∗^	268.70 ± 176.92^▲▲^	<0.001
Albumin (g/L)	47.20 (29.40, 53.40)	37.90 ± 7.21^∗^	32.61 ± 17.30	<0.001
Prothrombin activity (%)	79.00 (75, 118.00)	77.90 (29.30, 124.00)	35.00 (13.00, 39.50)^▲▲^	<0.001
International normalized ratio	1.02 (0.89, 1.29)	1.16 (0.87, 2.07)	2.01 (1.49, 5.32)^▲▲^	<0.001
White blood cell count (×10^9^/L)	5.98 ± 4.49	6.00 ± 3.27	7.98 ± 2.82^▲^	0.016
Neutrophil count (×10^9^/L)	2.71 (2.25, 10.67)	3.4 (0.86, 21.29)	5.81 (0.81, 72.31)^▲^	<0.001
Lymphocyte count (×10^9^/L)	1.82 (1.07, 3.31)	1.84 ± 0.56	1.09 ± 1.77^▲^	<0.001
NLR	1.85 (0.83, 7.46)	2.31 ± 2.98	4.05 ± 3.75^▲▲^	<0.001
Platelet count (×10^9^/L)	239.41 ± 49.92	126.46 ± 59.50^∗^	101.87 ± 49.31	0.031
Serum creatinine (mg/dL)	57.00 (42.00, 84.00)	68.00 (28.00, 108.00)	62.80 (33.00, 197.40)	0.152
Clusterin (*μ*g/mL)	213.45 (144.57, 313.48)	188.56 (70.22, 325.36)^∗∗^	51.09 (12.17, 188.62)^▲▲▲^	<0.001
*Underlying disease*				
Chronic hepatitis, *n* (%)	—	44 (69.84%)	72 (66.67%)	0.209
Liver cirrhosis, *n* (%)	—	19 (30.16%)	36 (33.33%)	0.161
Hepatitis B e antigen (+), *n* (%)	—	58 (92.06%)	103 (95.37%)	0.230
HBV DNA (log_10_ IU·mL-1)	—	11.90 ± 4.33	11.25 ± 3.89	0.365
MELD score	—	9.62 ± 8.65	27.74 ± 10.21^▲▲^	**0.003**
*Precipitating events*				
Spontaneous clearance of HBV	—	61 (96.83%)	103 (95.37%)	0.131
NUC cessation	—	2 (3.18%)	5 (4.63%)	0.205
NUC resistance	—	0 (0.0)	0 (0.0)	—
Hormone or immunosuppressive drugs	—	0 (0.0)	0 (0.0)	—
*Complications*				
Ascites, *n* (%)	—	19 (30.15%)	35 (32.41%)	0.064
Hepatic encephalopathy, *n* (%)	—	5 (7.93%)	29 (26.85%)^▲▲^	**0.003**
Spontaneous bacterial peritonitis, *n* (%)	—	15 (23.81%)	31 (28.70%)	0.190
Hyponatremia, *n* (%)	—	15 (23.51%)	33 (30.56%)	0.325
Gastrointestinal bleeding, *n* (%)	—	0 (0.0)	0 (0.0)	—
Hepatorenal syndrome, *n* (%)	—	2 (3.17%)	9 (8.33%)^▲^	0.021

Data are presented as *n* (%), mean ± SD, or median (minimum, maximum). Bold *P* values are significant at *P* < 0.05. HBV-non-ACLF vs. normal controls: ^∗^*P* < 0.05, ^∗∗^*P* < 0.01, and ^∗∗∗^*P* < 0.001. HBV-ACLF vs. HBV-non-ACLF: ^▲^*P* < 0.05, ^▲▲^*P* < 0.01, and ^▲▲▲^*P* < 0.001. Abbreviations: HBV-ACLF: hepatitis B virus-related acute-on-chronic liver failure; NLR: neutrophil-lymphocyte ratio; NUC: nucleos(t)ide analog.

**Table 2:** Association between serum clusterin level and clinical parameters in patients with hepatitis B virus-related acute-on-chronic liver failure.

Patient's parameters	Number of cases	Clusterin (*μ*g/mL)	*P* value
Age (years)			0.608
≤55	90	61.14 ± 40.58	
>55	18	66.54 ± 40.66	
Gender			0.075
Female	23	52.26 ± 23.89	
Male	85	64.69 ± 43.61	
Pathological base			0.489
Chronic hepatitis	72	58.90 ± 28.59	
Liver cirrhosis	36	46.14 ± 26.25	
Infection			0.478
With	31	60.05 ± 42.14	
Without	77	65.73 ± 38.23	
HBV DNA (log_10_ IU/mL)			0.388
≤10	41	57.73 ± 44.85	
>10	67	64.69 ± 37.63	
Serum creatinine (mg/dL)			0.566
≤1.50	99	51.09 (12.17, 231.86)	
>1.50	9	65.67 (20.36, 188.62)	
TBIL (mg/dL)			0.518
5-10 mg/dL	20	66.94 ± 9.77	
10-15 mg/dL	34	65.58 ± 6.72	
≥15 mg/dL	54	62.01 ± 5.06	
HBV-ACLF stages			**<0.001** ^∗^
30% < PTA ≤ 40%	44	72.59 ± 43.23	
20% < PTA ≤ 30%	44	46.03 ± 24.79	
PTA ≤ 20%	20	29.46 ± 12.74	
90-day outcome			**<0.001**
Survival	74	72.26 ± 43.52	
Nonsurvival	34	39.82 ± 19.34	
Survival time of the deaths (days)			**0.013**
≤28	21	28.39 (12.62, 68.96)	
>28	13	43.22 (28.01, 69.96)	

Data are presented as *n* (%), mean ± SD, or median (minimum, maximum). Abbreviations: HBV DNA: hepatitis B virus DNA; PTA: prothrombin activity; HBV-ACLF: hepatitis B virus-related acute-on-chronic liver failure. ^∗^Comparison among groups by one-way ANOVA. *P* values in bold numerals indicate significant values.

**Table 3:** Multivariate analysis of clinical variables for identifying hepatitis B virus-related acute-on-chronic liver failure.

Variable	*B* value	Standard error	*P* value	Odds ratio	95% CI
PTA (%)	0.336	0.113	**0.003**	1.399	(1.122, 1.745)
Clusterin (*μ*g/mL)	0.052	0.018	**0.004**	1.053	(1.017, 1.092)
TBIL (mg/dL)	-0.290	0.121	**0.016**	0.748	(0.590, 0.948)

*P* values in bold numerals indicate significant values. Abbreviations: PTA: prothrombin activity; TBIL: total bilirubin; CI: confidence intervals.

**Table 4:** Comparison of the area under the receiver operating characteristic curve, sensitivity, and specificity by the receiver operating characteristic curve for identifying hepatitis B virus-related acute-on-chronic liver failure.

Variable	AUC	95% CI	Cutoff value	Sensitivity	Specificity
PTA (%)	0.975	(0.000, 0.050)	40%	98.4%	65.8%
INR	0.976	(0.954, 0.998)	1.5	94.4%	88.9%
Total bilirubin (mg/dL)	0.888	(0.828, 0.948)	5.0	94.8%	69.8%
Clusterin (*μ*g/mL)	0.964	(0.011, 0.061)	105	93.7%	90.7%

Abbreviations: AUC: area under the receiver operating characteristic curve; PTA: prothrombin activity; INR: international normalized ratio; CI: confidence intervals.

**Table 5:** Univariate analysis of baseline clinical variables for hepatitis B virus-related acute-on-chronic liver failure survival and nonsurvival.

Characteristics	Survival (*n* = 74)	Nonsurvival (*n* = 34)	*P* value
Age (years)	42.66 ± 10.93	49.41 ± 8.50	**0.002**
Male (%), *n*	87.78% (62)	67.65% (23)	0.622
Alanine aminotransferase (U/L)	384.25 (85.70, 1863.20)	339.25 (29.70, 1834.40)	0.432
Aspartate aminotransferase (U/L)	391.15 (87.80, 1191.40)	332.30 (33.40, 1520.70)	0.718
Total bilirubin (mg/dL)	14.35 ± 5.54	18.62 ± 8.64	**0.011**
Albumin (g/L)	31.35 (22.00, 446.10)	30.45 (17.30, 248.00)	0.184
Serum creatinine (mg/dL)	0.70 (0.43, 1.64)	0.72 (0.37, 2.23)	0.794
Prothrombin activity (%)	36.97 ± 8.78	29.22 ± 10.34	**<0.001**
International normalized ratio	2.04 ± 0.53	2.4 ± 0.78	**<0.001**
Hepatitis B virus DNA (log IU/mL)	11.19 ± 4.05	11.40 ± 4.21	0.352
White blood cell (×10^9^/L)	6.17 ± 3.2.47	6.58 ± 3.49	0.538
Neutrophil count (×10^9^/L)	3.63 (0.81, 13.69)	4.09 (0.85, 72.31)	0.150
Lymphocyte count (×10^9^/L)	1.31 (0.31, 4.05)	1.00 (0.29, 18.22)	0.126
Neutrophil-lymphocyte ratio	3.60 ± 2.94	5.04 ± 5.00	**0.029**
Platelet count (×10^9^/L)	94.65 ± 47.56	86.56 ± 52.72	0.112
MELD score	20.78 ± 3.95	23.82 ± 4.50	**0.001**
Clusterin (*μ*g/mL)	62.78 (12.17, 231.86)	34.80 (12.62, 92.49)	**<0.001**
Spontaneous bacterial peritonitis, *n* (%)	19 (25.68%)	12 (35.29%)	0.067
Hepatic encephalopathy, *n* (%)	13 (17.57%)	16 (47.06%)	**0.010**
Hepatorenal syndrome, *n* (%)	3 (4.05%)	6 (17.65%)	**0.011**

Data are presented as *n* (%), mean ± SD, or median (minimum, maximum). *P* values in bold numerals indicate significant values. Abbreviations: MELD score: model for end-stage liver disease score.

**Table 6:** Multivariate analysis for predicting prognosis of patients with hepatitis B virus-related acute-on-chronic liver failure.

Variable	*B* value	Standard error	Odds ratio	95% CI
Age	-0.074	0.028	0.928	(0.879, 0.981)
Total bilirubin (mg/dL)	-0.106	0.042	0.900	(0.828, 0.977)
Clusterin (*μ*g/mL)	0.041	0.012	1.042	(1.017, 1.068)
Hepatic encephalopathy	1.054	0.545	2.869	(0.985, 8.354)

Abbreviations: CI: confidence intervals.

## Data Availability

The data was attached to supplemental files.

## Supplementary Materials

Supplementary Materials All original data of laboratory test results and demographic characteristics of included patients.
