# Supplementary material for: Serum Clusterin: A Potential Marker for Assessing the Clinical Severity and Short-Term Prognosis of Hepatitis B Virus-Related Acute-on-Chronic Liver Failure
Source: Dis Markers. 2020 Dec 12;2020:8814841. doi: 10.1155/2020/8814841 (PMC7755493; doi:10.1155/2020/8814841)
Supplement: Supplementary Materials — All original data of laboratory test results and demographic characteristics of included patients. [file 8814841.f1.pdf]

groups：1 HBV-ACL	number	ID	outcome（survival 1,	survival time	gender（female 1,	age
1.00	1.00	400822.00	1.00	31.00	2.00	53.00
1.00	2.00	845286.00	1.00	41.00	1.00	60.00
1.00	3.00	876085.00	0.00	15.00	2.00	53.00
1.00	4.00	877143.00	1.00	50.00	2.00	45.00
1.00	5.00	879105.00	1.00	39.00	2.00	37.00
1.00	6.00	160796.00	1.00	11.00	2.00	30.00
1.00	7.00	396708.00	1.00	24.00	2.00	67.00
1.00	8.00	892052.00	1.00	8.00	2.00	27.00
1.00	9.00	901814.00	1.00	27.00	2.00	29.00
1.00	10.00	907502.00	1.00	10.00	2.00	36.00
1.00	11.00	911466.00	1.00	21.00	2.00	58.00
1.00	12.00	913685.00	1.00	21.00	2.00	25.00
1.00	13.00	926708.00	1.00	13.00	2.00	31.00
1.00	14.00	940395.00	0.00	34.00	2.00	47.00
1.00	15.00	932557.00	1.00	30.00	2.00	37.00
1.00	16.00	955737.00	1.00	41.00	2.00	30.00
1.00	17.00	962306.00	1.00	22.00	2.00	37.00
1.00	18.00	961800.00	1.00	49.00	2.00	49.00
1.00	19.00	964698.00	1.00	21.00	2.00	37.00
1.00	20.00	253002.00	1.00	53.00	1.00	53.00
1.00	21.00	887168.00	1.00	29.00	2.00	56.00
1.00	22.00	467930.00	0.00	11.00	1.00	56.00
1.00	23.00	826056.00	0.00	22.00	1.00	36.00
1.00	24.00	684223.00	0.00	10.00	2.00	60.00
1.00	25.00	686107.00	0.00	15.00	2.00	38.00
1.00	26.00	678210.00	0.00	26.00	2.00	52.00
1.00	27.00	659753.00	1.00	20.00	1.00	43.00

1.00	28.00	733770.00	1.00	31.00	2.00	44.00
1.00	29.00	535008.00	1.00	41.00	2.00	47.00
1.00	30.00	470524.00	0.00	18.00	2.00	52.00
1.00	31.00	719053.00	1.00	25.00	2.00	40.00
1.00	32.00	722126.00	1.00	28.00	2.00	34.00
1.00	33.00	813570.00	1.00	78.00	2.00	52.00
1.00	34.00	803674.00	0.00	8.00	1.00	61.00
1.00	35.00	800521.00	0.00	14.00	1.00	41.00
1.00	36.00	824552.00	0.00	19.00	2.00	46.00
1.00	37.00	786739.00	0.00	16.00	2.00	43.00
1.00	38.00	195916.00	1.00	9.00	2.00	43.00
1.00	39.00	752699.00	0.00	27.00	2.00	41.00
1.00	40.00	782014.00	1.00	18.00	2.00	42.00
1.00	41.00	538336.00	1.00	26.00	1.00	36.00
1.00	42.00	86381.00	1.00	58.00	2.00	50.00
1.00	43.00	365541.00	1.00	28.00	2.00	37.00
1.00	44.00	678823.00	1.00	33.00	2.00	62.00
1.00	45.00	124278.00	1.00	26.00	2.00	27.00
1.00	46.00	238932.00	1.00	38.00	2.00	35.00
1.00	47.00	451232.00	0.00	36.00	1.00	55.00
1.00	48.00	127034.00	0.00	12.00	1.00	55.00
1.00	49.00	330506.00	0.00	35.00	2.00	40.00
1.00	50.00	187616.00	0.00	11.00	2.00	50.00
1.00	51.00	519676.00	0.00	68.00	2.00	43.00
1.00	52.00	536208.00	0.00	69.00	1.00	689.00
1.00	53.00	545405.00	0.00	84.00	2.00	38.00
1.00	54.00	551229.00	0.00	37.00	2.00	48.00
1.00	55.00	246617.00	0.00	48.00	1.00	49.00
1.00	56.00	553355.00	0.00	16.00	2.00	45.00
1.00	57.00	362356.00	0.00	26.00	2.00	70.00

1.00	58.00	268286.00	0.00	14.00	2.00	44.00
1.00	59.00	608160.00	0.00	21.00	1.00	48.00
1.00	60.00	622780.00	0.00	49.00	2.00	49.00
1.00	61.00	629446.00	0.00	16.00	2.00	49.00
1.00	62.00	305234.00	0.00	57.00	1.00	61.00
1.00	63.00	381260.00	0.00	46.00	2.00	37.00
1.00	64.00	324371.00	0.00	61.00	1.00	54.00
1.00	65.00	353674.00	0.00	35.00	2.00	42.00
1.00	66.00	330119.00	0.00	26.00	2.00	53.00
1.00	67.00	409177.00	0.00	9.00	2.00	56.00
1.00	68.00	441758.00	1.00	44.00	2.00	45.00
1.00	69.00	443336.00	1.00	29.00	2.00	40.00
1.00	70.00	443824.00	1.00	23.00	2.00	38.00
1.00	71.00	444606.00	1.00	25.00	2.00	42.00
1.00	72.00	450819.00	1.00	34.00	1.00	40.00
1.00	73.00	476389.00	1.00	39.00	1.00	49.00
1.00	74.00	513934.00	1.00	24.00	2.00	52.00
1.00	75.00	520924.00	1.00	29.00	2.00	30.00
1.00	76.00	538866.00	1.00	19.00	2.00	21.00
1.00	77.00	552778.00	1.00	31.00	2.00	65.00
1.00	78.00	565982.00	1.00	27.00	2.00	34.00
1.00	79.00	589720.00	1.00	52.00	2.00	44.00
1.00	80.00	595681.00	1.00	29.00	2.00	46.00
1.00	81.00	598796.00	1.00	30.00	1.00	39.00
1.00	82.00	601295.00	1.00	30.00	2.00	30.00
1.00	83.00	14945.00	1.00	25.00	2.00	54.00
1.00	84.00	617105.00	1.00	22.00	2.00	47.00
1.00	85.00	618275.00	1.00	27.00	2.00	44.00
1.00	86.00	553491.00	1.00	24.00	2.00	34.00
1.00	87.00	553491.00	1.00	26.00	2.00	34.00

1.00	88.00	558905.00	1.00	38.00	2.00	45.00
1.00	89.00	391919.00	1.00	16.00	2.00	26.00
1.00	90.00	378214.00	1.00	27.00	1.00	59.00
1.00	91.00	0000475966（2013	1.00	28.00	1.00	58.00
1.00	92.00	443998（2015-09-	1.00	20.00	2.00	44.00
1.00	93.00	503624.00	1.00	28.00	2.00	59.00
1.00	94.00	586608（2014-06-	1.00	9.00	1.00	44.00
1.00	95.00	287259.00	1.00	29.00	2.00	46.00
1.00	96.00	337661.00	1.00	35.00	2.00	69.00
1.00	97.00	426323.00	1.00	16.00	2.00	37.00
1.00	98.00	451232.00	1.00	35.00	1.00	60.00
1.00	99.00	435690.00	1.00	27.00	2.00	35.00
1.00	100.00	402389.00	1.00	19.00	2.00	43.00
1.00	101.00	689416.00	1.00	28.00	2.00	44.00
1.00	102.00	733770.00	1.00	30.00	2.00	44.00
1.00	103.00	541853.00	1.00	19.00	2.00	30.00
1.00	104.00	722126（2015-7-1	1.00	27.00	2.00	34.00
1.00	105.00	468434	1.00	23.00	2.00	45.00
1.00	106.00	719053.00	2.00	25.00	2.00	40.00
1.00	107.00	417296.00	1.00	37.00	1.00	54.00
1.00	108.00	758933.00	1.00	31.00	2.00	23.00
2.00	1.00	879206.00	0.00	＞90 days	2.00	25.00
2.00	2.00	286872.00	0.00	＞90 days	2.00	49.00
2.00	3.00	182973.00	0.00	＞90 days	2.00	24.00
2.00	4.00	129968.00	0.00	＞90 days	2.00	27.00
2.00	5.00	235351.00	0.00	＞90 days	1.00	21.00
2.00	6.00	23682.00	0.00	＞90 days	1.00	49.00
2.00	7.00	10863.00	0.00	＞90 days	1.00	22.00
2.00	8.00	38652.00	0.00	＞90 days	2.00	27.00
2.00	9.00	230752.00	0.00	＞90 days	2.00	46.00

2.00	10.00	3467432.00	0.00	＞90 days	2.00	35.00
2.00	11.00	458032.00	0.00	＞90 days	2.00	43.00
2.00	12.00	578543.00	0.00	＞90 days	2.00	38.00
2.00	13.00	379875.00	0.00	＞90 days	2.00	58.00
2.00	14.00	207653.00	0.00	＞90 days	1.00	59.00
2.00	15.00	248654.00	0.00	＞90 days	1.00	24.00
2.00	16.00	456905.00	0.00	＞90 days	2.00	29.00
2.00	17.00	456965.00	0.00	＞90 days	2.00	32.00
2.00	18.00	4578433.00	0.00	＞90 days	2.00	27.00
2.00	19.00	6779544.00	0.00	＞90 days	2.00	30.00
2.00	20.00	976534.00	0.00	＞90 days	2.00	69.00
2.00	21.00	233575.00	0.00	＞90 days	2.00	21.00
2.00	22.00	225534.00	0.00	＞90 days	2.00	29.00
2.00	23.00	184979.00	0.00	＞90 days	2.00	28.00
2.00	24.00	184882.00	0.00	＞90 days	2.00	32.00
2.00	25.00	184058.00	0.00	＞90 days	2.00	48.00
2.00	26.00	185419.00	0.00	＞90 days	2.00	45.00
2.00	27.00	848729.00	0.00	＞90 days	2.00	40.00
2.00	28.00	422215.00	0.00	＞90 days	2.00	34.00
2.00	29.00	850174.00	0.00	＞90 days	1.00	25.00
2.00	30.00	832351.00	0.00	＞90 days	1.00	28.00
2.00	31.00	855089.00	0.00	＞90 days	2.00	34.00
2.00	32.00	854690.00	0.00	＞90 days	1.00	57.00
2.00	33.00	245234.00	0.00	＞90 days	2.00	47.00
2.00	34.00	858607.00	0.00	＞90 days	2.00	41.00
2.00	35.00	531664.00	0.00	＞90 days	2.00	29.00
2.00	36.00	865189.00	0.00	＞90 days	2.00	49.00
2.00	37.00	860341.00	0.00	＞90 days	2.00	41.00
2.00	38.00	867912.00	0.00	＞90 days	1.00	46.00
2.00	39.00	884788.00	0.00	＞90 days	2.00	39.00

2.00	40.00	471778.00	0.00	＞90 days	2.00	35.00
2.00	41.00	835416.00	0.00	＞90 days	2.00	27.00
2.00	42.00	721313.00	0.00	＞90 days	1.00	21.00
2.00	43.00	179143.00	0.00	＞90 days	2.00	22.00
2.00	44.00	840355.00	0.00	＞90 days	2.00	28.00
2.00	45.00	538336.00	0.00	＞90 days	2.00	36.00
2.00	46.00	736914.00	0.00	＞90 days	2.00	34.00
2.00	47.00	760286.00	0.00	＞90 days	1.00	29.00
2.00	48.00	660074.00	0.00	＞90 days	2.00	35.00
2.00	49.00	401310.00	0.00	＞90 days	2.00	49.00
2.00	50.00	431787.00	0.00	＞90 days	2.00	77.00
2.00	51.00	445868.00	0.00	＞90 days	2.00	33.00
2.00	52.00	445092.00	0.00	＞90 days	2.00	69.00
2.00	53.00	416300.00	0.00	＞90 days	2.00	51.00
2.00	54.00	594903.00	0.00	＞90 days	2.00	49.00
2.00	55.00	457742.00	0.00	＞90 days	2.00	41.00
2.00	56.00	412324.00	0.00	＞90 days	2.00	43.00
2.00	57.00	453739	0.00	＞90 days	2.00	31.00
2.00	58.00	597810.00	0.00	＞90 days	2.00	27.00
2.00	59.00	407768	0.00	＞90 days	2.00	38.00
2.00	60.00	139059.00	0.00	＞90 days	2.00	36.00
2.00	61.00	210784.00	0.00	＞90 days	2.00	34.00
2.00	62.00	458232.00	0.00	＞90 days	2.00	57.00
2.00	63.00	458278.00	0.00	＞90 days	2.00	36.00
3.00	1.00	1.00	0.00	＞90 days	1.00	47.00
3.00	2.00	2.00	0.00	＞90 days	1.00	40.00
3.00	3.00	3.00	0.00	＞90 days	1.00	31.00
3.00	4.00	4.00	0.00	＞90 days	1.00	30.00
3.00	5.00	5.00	0.00	＞90 days	1.00	29.00
3.00	6.00	6.00	0.00	＞90 days	1.00	28.00

3.00	7.00	7.00	0.00	＞90 days	2.00	44.00
3.00	8.00	8.00	0.00	＞90 days	1.00	31.00
3.00	9.00	9.00	0.00	＞90 days	1.00	32.00
3.00	10.00	10.00	0.00	＞90 days	1.00	79.00
3.00	11.00	11.00	0.00	＞90 days	1.00	32.00
3.00	12.00	12.00	0.00	＞90 days	1.00	48.00
3.00	13.00	13.00	0.00	＞90 days	1.00	31.00
3.00	14.00	14.00	0.00	＞90 days	1.00	53.00
3.00	15.00	15.00	0.00	＞90 days	1.00	25.00
3.00	16.00	16.00	0.00	＞90 days	1.00	41.00
3.00	17.00	17.00	0.00	＞90 days	1.00	29.00
3.00	18.00	18.00	0.00	＞90 days	1.00	48.00
3.00	19.00	19.00	0.00	＞90 days	1.00	54.00
3.00	20.00	20.00	0.00	＞90 days	2.00	33.00
3.00	21.00	21.00	0.00	＞90 days	2.00	60.00
3.00	22.00	22.00	0.00	＞90 days	2.00	41.00
3.00	23.00	23.00	0.00	＞90 days	1.00	29.00
3.00	24.00	24.00	0.00	＞90 days	1.00	84.00
3.00	25.00	25.00	0.00	＞90 days	1.00	29.00
3.00	26.00	26.00	0.00	＞90 days	1.00	26.00
3.00	27.00	27.00	0.00	＞90 days	1.00	37.00
3.00	28.00	28.00	0.00	＞90 days	1.00	31.00
3.00	29.00	29.00	0.00	＞90 days	1.00	31.00
3.00	30.00	30.00	0.00	＞90 days	1.00	34.00
3.00	31.00	31.00	0.00	＞90 days	1.00	43.00
3.00	32.00	32.00	0.00	＞90 days	1.00	30.00
3.00	33.00	33.00	0.00	＞90 days	1.00	31.00
3.00	34.00	34.00	0.00	＞90 days	1.00	22.00
3.00	35.00	35.00	0.00	＞90 days	1.00	29.00
3.00	36.00	36.00	0.00	＞90 days	2.00	52.00

3.00	37.00	37.00	0.00	＞90 days	2.00	41.00
3.00	38.00	38.00	0.00	＞90 days	1.00	32.00
3.00	39.00	39.00	0.00	＞90 days	2.00	56.00
3.00	40.00	40.00	0.00	＞90 days	2.00	42.00
3.00	41.00	41.00	0.00	＞90 days	1.00	46.00
3.00	42.00	42.00	0.00	＞90 days	2.00	34.00
3.00	43.00	43.00	0.00	＞90 days	1.00	44.00
3.00	44.00	44.00	0.00	＞90 days	1.00	34.00

ALT0	AST0	TBIL0	TBIL(MG/DL)	ALB0	Cr0	Cr(mg/dl)	PTA0	INR0	AFP	HBVDNA
561.00	422.40	380.50	22.25	27.90	73.50	0.83	29.00	2.60	131.10	2750000.00
775.90	725.10	319.60	18.69	31.60	38.40	0.43	35.00	2.43	345.40	12600.00
1370.90	816.90	261.20	15.27	37.00	60.00	0.68	29.00	2.77	18.20	32300000.00
461.40	531.30	312.40	18.27	31.00	67.60	0.76	41.00	2.07	388.20	471000.00
265.80	547.20	330.70	19.34	31.00	50.00	0.57	44.00	1.78	307.00	232000.00
1543.10	933.00	230.80	13.50	30.60	60.00	0.68	20.00	3.91	11.30	47500.00
265.00	212.20	182.70	10.68	31.20	54.00	0.61	46.00	1.92	247.50	139000.00
105.10	87.80	364.30	21.30	31.50	49.90	0.56	33.00	2.48	77.30	1460.00
336.50	428.00	118.10	6.91	34.70	80.30	0.91	32.00	2.59		33200.00
387.50	413.60	192.40	11.25	26.20	76.20	0.86	27.00	2.92	988.90	4190000.00
381.00	225.00	195.00	11.40	35.40	81.30	0.92	39.00	2.16	29.40	729000.00
1057.40	258.00	109.10	6.38	85.10	81.30	0.92	45.00	1.95	25.50	28300.00
1018.40	570.90	387.00	22.63	33.30	66.00	0.75	45.00	1.94	4.97	52600000.00
578.00	720.00	228.00	13.33	27.50	70.70	0.80	30.00	2.68	5.00	3140.00
578.00	638.00	349.60	20.44	32.80	75.00	0.85	34.00	2.41	191.10	3210.00
293.00	392.70	222.20	12.99	32.40	58.60	0.66	37.00	2.30	105.30	3850.00
469.30	177.80	367.70	21.50	30.60	49.00	0.55	35.00	2.13	642.40	1530000.00
269.30	915.90	264.30	15.46	30.00	63.00	0.71	33.00	2.50	157.00	214.00
223.80	495.00	131.70	7.70	34.30	63.10	0.71	24.00	2.99	519.70	2050000.00
292.20	286.80	431.10	25.21	31.20	63.30	0.72	40.00	1.80	93.50	11900.00
731.00	463.00	153.20	8.96	22.00	96.20	1.09	21.00	3.67	2.00	100.00
657.00	623.00	239.20	13.99	30.50	58.00	0.66	28.00	2.67	22.50	190000000.00
139.90	250.10	226.80	13.26	30.40	65.10	0.74	40.00	2.10	143.80	575.00
569.00	623.80	701.60	41.03	31.50	197.40	2.23	42.00	1.96	163.90	500.00
713.80	651.00	330.00	19.30	183.30	48.00	0.54	16.80	2.94	16.20	6880000.00
101.70	111.10	340.20	19.89	248.00	61.50	0.70	42.50	1.64	418.90	1000.00
496.00	736.00	183.40	10.73	26.80	61.00	0.69	42.80	1.63	27.80	62700.00

232.70	297.40	192.90	11.28	35.10	61.00	0.69	35.00	2.21	33.60	1160000.00
212.40	404.10	400.90	23.44	29.60	73.00	0.83	37.70	1.77	369.40	1580.00
306.00	685.00	284.50	16.64	33.20	86.00	0.97	17.00	4.19	8.10	454000.00
294.80	133.60	199.70	11.68	28.50	60.00	0.68	39.00	2.01	36.00	556000.00
343.10	329.40	313.70	18.35	29.10	56.00	0.63	46.00	1.77	170.50	230000.00
968.80	743.00	218.70	12.79	31.80	53.00	0.60	23.00	3.16	33.10	20700000.00
100.70	121.10	383.30	22.42	26.80	45.60	0.52	31.00	2.46	134.30	224000.00
470.40	777.20	359.10	21.00	32.50	79.10	0.89	13.00	5.32	171.30	844000.00
103.80	192.80	691.40	40.43	38.50	49.90	0.56	26.00	3.06	97.70	13500.00
370.30	170.40	334.30	19.55	30.60	79.10	0.89	37.40	1.77	11.20	60100.00
1139.10	400.30	361.30	21.13	446.10	57.10	0.65	27.60	2.15	87.40	384000.00
409.00	751.00	495.10	28.95	33.60	43.00	0.49	24.90	2.29		135.00
1330.20	1191.40	149.30	8.73	38.40	75.30	0.85	39.80	1.71	37.70	21400.00
466.70	313.10	127.60	7.46	31.50	62.00	0.70	47.00	1.62	136.10	4.14
231.00	549.00	137.30	8.03	63.80	42.00	0.48	51.50	1.50	37.00	1.32
150.50	177.50	162.60	9.51	36.40	78.00	0.88	34.00	2.05		4660.00
322.00	177.70	280.40	16.40	27.00	62.90	0.71	43.90	1.60	114.80	4450.00
1105.00	649.10	287.60	16.82	32.20	60.00	0.68	21.00	3.04	70.70	208.00
422.60	894.40	297.20	17.38	28.30	82.40	0.93	31.00	2.20	37.40	500.00
85.70	124.90	266.50	15.58	29.90	72.00	0.81	51.50	1.50	16.80	99200.00
258.30	261.00	85.00	4.97	27.90	62.90	0.71	30.70	2.01	29.90	2670000.00
88.70	138.30	177.20	10.36	24.20	80.10	0.91	29.70	2.05	14.70	500.00
876.20	199.80	344.40	20.14	27.40	56.00	0.63	25.20	2.27	194.60	581000.00
1834.40	1338.60	287.90	16.84	34.00	65.10	0.74	30.40	2.02	11.50	1530000.00
331.80	326.40	237.10	13.87	23.20	54.00	0.61	19.00	3.33	26.40	627000.00
127.10	95.50	366.60	21.44	25.50	61.00	0.69	15.80	3.05	825.00	109.00
785.60	746.90	217.80	12.74	31.80	94.50	1.07	24.20	2.33	354.10	3860000.00
173.40	147.80	91.10	5.33	17.30	55.00	0.62	50.00	1.59	1.70	475000.00
191.70	353.90	351.10	20.53	29.60	91.50	1.04	26.30	2.07	1777.00	29400.00
776.90	867.90	88.30	5.16	28.50	115.00	1.30	23.10	2.40	5.50	1390000.00

652.00	672.00	543.30	31.77	30.90	138.00	1.56	43.00	1.73	3.40	1750.00
29.70	49.60	205.00	11.99	35.90	33.00	0.37	18.90	2.73	3.50	4500.00
1638.30	1520.70	362.80	21.22	30.90	48.70	0.55	14.30	3.25	13.50	1220000.00
343.80	500.30	472.20	27.61	30.00	70.50	0.80	30.00	2.04	39.70	528000.00
332.70	299.70	206.00	12.05	24.20	65.90	0.75	38.00	1.62	63.60	372000.00
334.70	203.70	315.40	18.44	28.50	52.00	0.59	46.60	1.55	302.00	2740.00
69.10	39.10	243.90	14.26	29.80	52.70	0.60	22.10	2.39	56.60	23200.00
423.30	338.20	426.20	24.92	30.60	42.30	0.48	32.30	1.91	146.90	21500.00
114.50	33.40	177.70	10.39	17.70	103.40	1.17	17.50	2.76	8.60	500.00
964.70	753.80	485.10	28.37	34.90	65.60	0.74	27.30	2.16	25.40	8090000000.00
262.00	288.30	298.10	17.43	34.20	61.70	0.70	35.80	1.83	25.70	500.00
1863.20	1080.70	110.70	6.47	36.10	72.80	0.82	26.10	2.22	61.30	156000.00
1547.00	532.20	274.00	16.02	36.00	60.20	0.68	29.70	2.05	166.20	529000.00
125.30	195.90	126.70	7.41	26.40	56.00	0.63	33.50	1.90	30.60	661000.00
295.60	267.90	124.72	7.29	33.20	47.40	0.54	31.00	2.35	34.10	185000000.00
463.60	281.60	321.30	18.79	27.40	68.00	0.77	33.30	1.91	531.80	25600.00
151.00	213.40	211.70	12.38	27.10	68.30	0.77	35.30	1.84	67.00	781000.00
283.00	266.90	355.90	20.81	30.00	76.90	0.87	42.80	1.63	182.50	37100.00
753.40	458.30	199.90	11.69	38.20	68.00	0.77	48.30	1.51	72.80	8970000.00
1002.00	627.00	444.30	25.98	28.80	61.70	0.70	28.00	2.13	112.20	465000.00
1120.20	684.00	154.00	9.01	33.70	61.30	0.69	48.30	1.51	20.10	28100000.00
232.40	125.70	262.30	15.34	38.90	47.60	0.54	22.00	2.93	12.40	148000.00
295.60	372.90	423.50	24.77	29.50	46.90	0.53	37.40	1.77	272..2	1040.00
777.70	1037.80	293.40	17.16	29.60	50.50	0.57	24.20	2.33	43.90	5250.00
804.50	969.90	171.00	10.00	32.80	62.80	0.71	24.50	2.32	138.70	147000.00
200.90	155.80	215.60	12.61	24.40	59.00	0.67	36.90	1.79	139.50	4380.00
237.40	205.90	346.90	20.29	26.10	145.00	1.64	14.30	3.25	74.30	500.00
901.90	611.20	286.90	16.78	30.60	48.00	0.54	31.10	2.00	251.40	123000.00
358.80	309.00	99.50	5.82	32.40	52.60	0.60	48.70	1.50	32.00	25900000.00
358.80	309.00	99.50	5.82	32.40	52.60	0.60	48.70	1.50	32.00	25900000.00

187.00	99.40	353.80	20.69	28.40	74.90	0.85	38.60	1.74	40.80	6130.00
1008.10	543.20	216.20	12.64	31.90	80.80	0.91	38.60	1.74	518.90	226000.00
433.80	683.40	204.00	11.93	31.50	48.40	0.55	43.00	1.49	126.30	24200.00
169.10	100.40	130.80	7.65	33.60	66.30	0.75	32.40	1.94	5,8	134000.00
484.00	514.00	215.00	12.57	31.70	59.00	0.67	45.40	1.57	4.00	100.00
436.00	556.00	191.20	11.18	33.00	143.60	1.62	51.00	1.57	5.50	500.00
496.30	348.00	208.50	12.19	28.10	53.00	0.60	23.50	2.38	124.80	23900000.00
472.00	389.60	341.50	19.97	27.80	70.80	0.80	42.80	1.63		500.00
395.90	486.70	165.50	9.68	27.90	125.00	1.41	39.80	1.71	>1000	467000.00
781.80	934.20	199.00	11.64	33.40	56.90	0.64	45.00	1.58	>1000	13100000.00
85.70	124.90	266.50	15.58	29.90	96.00	1.09	51.50	1.50	16.80	99200.00
575.20	347.80	436.40	25.52	34.20	67.60	0.76	51.00	1.56	163.10	500.00
263.10	243.30	165.60	9.68	29.90	62.90	0.71	45.00	1.55	1000.00	511000.00
272.70	296.90	310.60	18.16	239.80	64.00	0.72	33.60	1.62	225.90	267000000.00
132.70	197.40	192.90	11.28	35.10	51.00	0.58	42.50	1.64	33.60	1160000.00
162.60	163.60	107.40	6.28	28.80	54.00	0.61	41.00	1.78	12.80	500.00
668.00	729.60	334.90	19.58	28.90	60.80	0.69	45.80	1.56	170.50	794000.00
129.10	138.60	174.00	10.18	143.90	66.20	0.75	44.70	1.59	248.70	5200000.00
644.40	406.20	184.80	10.81	31.20	72.00	0.81	40.00	2.00	36.00	556000.00
232.30	166.90	252.50	14.77	25.90	54.90	0.62	38.00	1.75	69.40	767000.00
203.80	145.10	334.40	19.56	28.20	56.00	0.63	44.00	1.86	628.70	192000.00
220.80	168.80	31.60	1.85	40.60	55.00	0.62	100.00	1.00		2970000.00
482.70	382.20	160.00	9.36	32.20	108.00	1.22	117.00	0.89	31.10	23900000.00
583.50	77.50	33.50	1.96	42.50	69.00	0.78	109.00	1.00	19.90	10400000.00
352.20	160.00	32.80	1.92	38.00	64.00	0.72	76.00	1.21	14.80	26400000.00
170.20	231.90	79.00	4.62	43.50	64.00	0.72	104.00	0.97	2.70	38000.00
611.80	403.80	61.60	3.60	34.50	54.00	0.61	53.00	1.64	80.80	24900.00
122.00	144.00	69.00	4.04	39.30	65.00	0.74	79.00	1.12		414.00
96.20	57.80	22.20	1.30	15.80	65.00	0.74	124.00	0.87	46.00	405.00
130.40	127.00	11.60	0.68	45.20	72.00	0.81	109.00	0.94	5.00	100.00

130.40	127.00	11.60	0.68	45.20	72.00	0.81	109.00	0.94	5.00	100.00
365.40	307.10	53.90	3.15	32.30	71.00	0.80	53.00	1.59		2100000.00
365.40	307.10	53.90	3.15	32.30	71.00	0.80	53.00	1.59		2100000.00
470.60	131.60	29.70	1.74	38.50	86.00	0.97	77.00	1.20	8.30	48600000.00
154.00	178.00	99.00	5.79	49.80	68.00	0.77	99.00	1.01		100.00
331.30	121.60	23.50	1.37	40.40	69.00	0.78	74.00	1.24		96400000.00
1622.60	744.70	21.10	1.23	35.40	68.00	0.77	70.00	1.28	1.00	100.00
181.00	78.10	4.30	0.25	38.20	88.00	1.00	84.00	1.13		257000000.00
504.60	1714.90	40.50	2.37	50.00	58.20	0.66	96.00	1.03	81.40	24800.00
181.00	78.10	4.30	0.25	38.20	88.00	1.00	84.00	1.13		257000000.00
78.60	69.50	15.90	0.93	36.50	69.00	0.78	86.00	1.11	9.40	2000000.00
432.10	155.80	23.30	1.36	66.70	66.00	0.75	93.10	1.00	7.20	53000000.00
111.70	36.00	51.10	2.99	37.10	79.00	0.89	100.00	1.00		1810.00
476.40	119.60	7.70	0.45	38.60	72.00	0.81	103.00	0.98	11.00	1.16
416.30	114.60	11.50	0.67	32.60	68.00	0.77	81.00	1.16	18.40	193000000.00
80.00	52.40	40.30	2.36	46.90	47.40	0.54	94.40	0.99	5.20	100.00
141.90	854.20	210.60	12.32	36.80	74.10	0.84	91.00	1.13	17.20	100.00
383.30	457.90	276.10	16.15	35.40	61.20	0.69	75.00	1.29	182.80	7520.00
147.90	61.30	22.20	1.30	45.50	72.00	0.81	78.00	1.18	2.90	1830000.00
1868.70	1024.60	74.70	4.37	41.00	51.00	0.58	80.00	1.23	2.70	4690.00
239.00	186.90	17.20	1.01	43.90	48.00	0.54	97.00	1.02	20.60	3330.00
753.70	248.30	31.10	1.82	39.00	83.00	0.94	95.00	1.09		26200000.00
267.30	276.00	24.50	1.43	33.90	48.50	0.55	80.00	1.23	224.80	1590000.00
180.90	44.60	18.70	1.09	40.10	73.00	0.83	85.00	1.12	3.50	16800.00
55.00	26.20	12.40	0.73	44.50	80.00	0.90	107.00	0.95	2.60	603.00
103.50	46.60	16.90	0.99	48.50	74.00	0.84	82.80	1.08		11000000.00
861.60	1637.50	51.20	2.99	35.20	76.30	0.86	91.00	1.13	18.90	2360000.00
328.60	122.90	30.20	1.77	37.80	63.00	0.71	93.00	1.11	25.20	237000.00
115.40	52.30	22.00	1.29	40.20	47.00	0.53	85.00	1.12	1.60	65400.00
662.70	376.30	40.00	2.34	36.70	66.00	0.75	92.00	1.05	35.80	13900000.00

675.70	1490.00	71.80	4.20	41.60	64.20	0.73	71.70	1.18	69.20	9860000.00
342.70	686.40	40.90	2.39	41.00	69.70	0.79	106.00	1.02	43.30	523000.00
138.00	78.40	11.30	0.66	38.60	55.00	0.62	82.80	1.08	8.30	25600000.00
64.20	37.00	17.60	1.03	49.50	51.00	0.58	66.30	1.16		25200000.00
319.20	136.50	370.20	21.65	37.50	75.40	0.85	63.00	1.46	394.60	3770.00
745.90	379.00	67.50	3.95	31.50	53.00	0.60	47.00	1.62	98.60	410000.00
434.40	192.90	134.20	7.85	32.30	72.80	0.82	61.00	1.42	341.20	2060000.00
550.60	1469.30	270.20	15.80	35.20	28.00	0.32	62.10	1.29	87.60	42000.00
1193.40	349.60	118.10	6.91	35.10	84.00	0.95	72.00	1.21	350.00	8790.00
1239.20	287.50	182.10	10.65	31.70	64.10	0.73	44.70	1.59	112.90	6140000.00
219.40	119.50	123.80	7.24	36.00	77.20	0.87	56.00	1.37	57.10	32900.00
120.20	89.00	395.90	23.15	28.90	63.10	0.71	64.80	1.26	189.90	500.00
64.00	72.30	192.30	11.25	32.40	71.50	0.81	46.00	1.34	7.30	500.00
530.30	346.30	68.50	4.01	36.10	72.00	0.81	76.90	1.13	48.80	623000000.00
268.20	196.30	112.70	6.59	36.60	62.00	0.70	62.00	1.33	443.00	500.00
753.30	538.30	82.80	4.84	29.70	74.00	0.84	29.30	2.07	380.90	1050000.00
78.10	60.60	50.20	2.94	31.90	62.00	0.70	59.00	1.33	34.90	10000.00
85.00	99.70	383.60	22.43	31.60	51.80	0.59	45.00	1.36	81.20	500.00
914.00	948.60	88.10	5.15	38.00	67.00	0.76	57.00	1.40	17.10	6050000.00
402.80	155.80	275.80	16.13	35.00	74.00	0.84	57.80	1.35	616.00	14800.00
97.00	91.90	316.60	18.51	29.40	38.00	0.43	70.80	1.19	319.30	274000.00
797.90	305.70	30.20	1.77	40.60	84.00	0.95	44.00	1.39	4.20	139000.00
109.60	153.60	138.20	8.08	21.20	104.00	1.18	47.00	1.54		2540000.00
984.80	1262.40	252.90	14.79	38.10	55.00	0.62	57.00	1.49	46.00	7350000.00
18.80	21.40	11.80	0.69	47.40	60.00	0.68	NA	NA	NA	NA
15.00	16.50	10.30	0.60	47.70	74.00	0.84	NA	NA	NA	NA
28.10	29.40	17.80	1.04	49.00	69.00	0.78	NA	NA	NA	NA
8.40	11.70	6.30	0.37	46.30	46.00	0.52	NA	NA	NA	NA
13.30	12.20	5.90	0.35	48.40	54.00	0.61	NA	NA	NA	NA
18.60	16.90	8.70	0.51	46.70	56.00	0.63	NA	NA	NA	NA

69.40	41.50	13.10	0.77	52.40	71.00	0.80	NA	NA	NA	NA
13.80	16.40	9.20	0.54	49.10	61.00	0.69	NA	NA	NA	NA
36.20	22.20	8.70	0.51	43.70	43.00	0.49	NA	NA	NA	NA
33.70	27.70	8.00	0.47	45.50	57.00	0.64	NA	NA	NA	NA
8.90	17.00	9.90	0.58	51.10	57.00	0.64	NA	NA	NA	NA
31.50	22.40	5.90	0.35	46.00	50.00	0.57	NA	NA	NA	NA
8.30	15.90	13.60	0.80	50.80	52.00	0.59	NA	NA	NA	NA
42.60	23.80	8.30	0.49	48.10	67.00	0.76	NA	NA	NA	NA
10.00	16.40	7.20	0.42	49.70	56.00	0.63	NA	NA	NA	NA
11.50	13.90	10.30	0.60	44.20	53.00	0.60	NA	NA	NA	NA
19.50	18.20	8.20	0.48	47.20	47.00	0.53	NA	NA	NA	NA
10.70	15.70	12.80	0.75	46.60	59.00	0.67	NA	NA	NA	NA
17.50	15.50	7.30	0.43	49.80	56.00	0.63	NA	NA	NA	NA
13.10	11.90	8.00	0.47	51.80	57.00	0.64	NA	NA	NA	NA
23.40	24.70	14.00	0.82	48.70	77.00	0.87	NA	NA	NA	NA
47.20	78.00	25.60	1.50	46.40	76.00	0.86	NA	NA	NA	NA
12.00	17.50	10.50	0.61	50.50	60.00	0.68	NA	NA	NA	NA
13.50	13.90	18.50	1.08	53.40	50.00	0.57	NA	NA	NA	NA
10.70	13.50	13.00	0.76	47.10	44.00	0.50	NA	NA	NA	NA
9.20	13.90	10.50	0.61	46.90	61.00	0.69	NA	NA	NA	NA
15.70	13.50	9.40	0.55	50.70	50.00	0.57	NA	NA	NA	NA
11.70	17.60	16.00	0.94	48.70	69.00	0.78	NA	NA	NA	NA
24.50	16.90	5.80	0.34	46.90	42.00	0.48	NA	NA	NA	NA
11.50	13.30	59.00	3.45	42.70	48.00	0.54	NA	NA	NA	NA
24.00	19.60	12.70	0.74	41.80	63.00	0.71	NA	NA	NA	NA
24.00	19.60	12.70	0.74	41.80	63.00	0.71	NA	NA	NA	NA
11.80	13.90	10.20	0.60	47.20	50.00	0.57	NA	NA	NA	NA
9.40	11.70	23.50	1.37	47.30	55.00	0.62	NA	NA	NA	NA
11.50	12.20	8.30	0.49	46.40	49.00	0.55	NA	NA	NA	NA
32.70	26.30	7.00	0.41	46.40	60.00	0.68	NA	NA	NA	NA

23.10	18.20	6.50	0.38	48.30	80.00	0.90	NA	NA	NA	NA
19.20	16.90	7.90	0.46	45.70	61.00	0.69	NA	NA	NA	NA
17.40	23.00	11.90	0.70	47.10	73.00	0.83	NA	NA	NA	NA
35.90	20.20	17.80	1.04	29.40	84.00	0.95	NA	NA	NA	NA
10.70	16.20	3.70	0.22	46.90	53.00	0.60	NA	NA	NA	NA
16.10	16.10	13.90	0.81	51.10	71.00	0.80	NA	NA	NA	NA
9.80	12.50	15.60	0.91	50.70	52.00	0.59	NA	NA	NA	NA
8.90	12.00	7.00	0.41	46.70	51.00	0.58	NA	NA	NA	NA

HBVDNA-LOG	WBC	NC	LC	NLR	PLT	CLU(ug/ml_)
14.83	4.32	2.95	0.82	3.60	20.10	30.60
9.44	2.82	2.11	0.41	5.15	61.40	67.03
17.29	5.50	3.80	0.90	4.22	96.00	54.27
13.06	6.16	3.90	0.79	4.94	141.00	86.34
12.35	6.90	3.60	2.70	1.33	123.00	100.64
10.77	6.92	2.15	4.05	0.53	131.00	40.15
11.84	3.89	2.86	0.82	3.49	100.00	89.12
7.29	3.35	1.66	1.36	1.22	71.00	59.92
10.41	5.62	3.65	1.26	2.90	166.00	80.92
15.25	17.21	13.69	2.39	5.73	67.00	70.97
13.50	4.91	1.58	2.41	0.66	50.00	86.34
10.25	6.53	3.98	1.65	2.41	131.00	69.42
17.78	8.32	5.10	1.88	2.71	121.40	62.80
8.05	2.27	1.34	0.66	2.03	30.40	32.07
8.07	4.28	3.07	0.78	3.94	87.00	50.39
8.26	6.70	2.78	3.11	0.89	76.00	53.55
14.24	9.45	5.80	2.28	2.54	110.40	46.72
5.37	2.32	0.88	0.95	0.93	65.40	38.33
14.53	4.82	2.80	1.38	2.03	66.40	37.58
9.38	3.61	2.74	0.52	5.27	65.00	51.09
4.61	9.83	7.79	0.76	10.25	19.40	30.12
19.06	16.60	14.90	1.20	12.42	62.00	49.76
6.35	7.41	4.53	2.17	2.09	175.40	30.15
6.21	14.16	12.85	0.44	29.20	142.40	90.99
15.74	5.65	3.69	1.18	3.13	85.80	16.22
6.91	2.74	2.21	0.29	7.62	46.40	50.82
11.05	3.65	1.70	1.02	1.67	70.00	35.69

13.96	8.74	7.17	0.98	7.32	134.30	29.95
7.37	6.44	4.84	1.18	4.10	90.00	62.27
13.03	6.30	3.10	2.20	1.41	45.00	12.62
13.23	3.70	2.10	1.00	2.10	43.00	40.84
12.35	5.00	3.50	0.80	4.38	125.00	35.30
16.85	4.03	1.77	1.77	1.00	93.00	42.33
12.32	8.72	7.13	0.88	8.10	76.00	38.35
13.65	5.58	3.33	1.47	2.27	117.40	20.22
9.51	4.64	3.05	1.02	2.99	100.00	25.44
11.00	7.56	4.95	1.41	3.51	82.60	21.95
12.86	10.19	8.71	0.77	11.31	292.00	18.82
4.91	2.79	1.94	0.49	3.96	31.40	25.75
9.97	5.81	3.82	1.45	2.63	135.00	18.32
1.42	5.38	2.73	1.79	1.53	133.50	30.68
0.28	1.58	0.81	0.53	1.53	47.20	18.58
8.45	3.35	2.61	0.49	5.33	28.20	20.80
8.40	5.96	4.36	0.90	4.84	96.40	29.10
5.34	8.41	4.78	2.47	1.94	112.40	12.17
6.21	6.28	3.47	2.11	1.64	74.40	18.24
11.50	6.49	5.23	0.54	9.69	21.80	69.96
14.80	4.67	2.90	1.10	2.64	25.60	23.62
6.21	8.76	6.68	1.15	5.81	43.40	39.53
13.27	14.83	11.01	3.42	3.22	162.00	28.22
14.24	5.33	4.16	0.86	4.84	122.40	42.29
13.35	6.45	4.82	0.96	5.02	47.40	33.34
4.69	7.34	5.69	0.84	6.77	111.00	53.07
15.17	3.39	2.34	0.76	3.08	97.00	65.67
13.07	2.60	1.17	1.08	1.08	31.10	28.01
10.29	6.23	4.14	1.26	3.29	109.00	20.36
14.14	6.99	5.40	0.83	6.51	144.00	28.39

7.47	5.85	4.12	0.95	4.34	41.70	92.49
8.41	1.51	0.85	0.47	1.81	20.80	31.47
14.01	7.85	72.31	18.22	3.97	113.00	35.08
13.18	4.70	4.05	0.44	9.20	133.00	22.13
12.83	4.94	2.99	1.05	2.85	75.00	52.42
7.92	11.41	7.42	2.72	2.73	90.40	44.73
10.05	4.14	2.48	0.98	2.53	35.40	43.22
9.98	9.72	6.77	2.00	3.39	198.00	61.49
6.21	6.04	3.97	1.27	3.13	22.00	34.53
22.81	4.57	2.35	0.98	2.40	208.30	35.26
6.21	3.74	2.23	0.87	2.56	101.40	53.32
11.96	5.75	3.84	1.16	3.31	155.00	130.05
13.18	6.53	3.93	2.19	1.79	81.00	62.77
13.40	9.18	5.32	2.87	1.85	74.30	73.79
19.04	3.57	2.81	0.59	4.76	99.40	69.70
10.15	12.23	8.71	2.32	3.75	165.70	93.20
13.57	5.72	3.10	1.98	1.57	73.00	107.89
10.52	6.07	3.99	1.57	2.54	123.40	86.85
16.01	5.78	3.40	1.59	2.14	178.00	56.60
13.05	5.41	4.52	0.39	11.59	95.00	46.92
17.15	7.37	4.62	1.90	2.43	115.00	64.05
11.90	7.64	4.72	2.21	2.14	60.40	46.92
6.95	7.29	5.37	1.20	4.48	99.00	62.70
8.57	6.23	4.48	1.22	3.67	56.30	63.58
11.90	8.07	4.76	1.98	2.40	191.10	43.24
8.38	6.73	4.88	1.09	4.48	40.40	41.12
6.21	11.92	7.79	2.17	3.59	61.00	35.16
11.72	5.44	2.92	1.83	1.60	92.00	48.43
17.07	6.36	3.95	1.53	2.58	79.10	60.65
17.07	6.36	3.95	1.53	2.58	79.10	100.33

8.72	6.19	3.08	2.08	1.48	38.40	74.88
12.33	5.69	3.56	1.59	2.24	97.00	80.28
10.09	5.62	3.47	1.65	2.10	74.40	39.30
11.81	3.96	1.70	1.63	1.04	42.80	87.91
4.61	3.50	2.50	0.50	5.00	28.00	170.43
6.21	3.33	2.08	0.88	2.36	55.40	103.46
16.99	8.24	5.08	2.07	2.45	148.80	88.79
6.21	5.86	4.72	0.66	7.15	107.00	231.86
13.05	5.21	3.98	0.79	5.04	48.40	188.62
16.39	5.43	3.72	1.22	3.05	93.00	169.17
11.50	6.09	5.43	0.31	17.52	28.40	101.39
6.21	7.83	5.90	1.11	5.32	102.00	171.56
13.14	5.07	3.21	1.45	2.21	73.00	97.68
19.40	9.09	7.91	0.64	12.36	196.00	139.55
13.96	8.74	1.17	0.98	1.19	134.30	64.93
6.21	4.87	3.60	0.70	5.14	31.90	103.21
13.58	4.80	3.32	0.80	4.15	161.00	101.09
15.46	4.78	3.16	1.06	2.98	116.00	146.89
13.23	3.99	1.89	1.56	1.21	56.40	97.60
13.55	7.65	5.14	1.79	2.87	88.40	53.21
12.17	6.40	3.90	1.80	2.17	117.00	123.08
14.90	4.88	2.81	1.70	1.65	247.10	212.24
16.99	7.38	3.94	2.06	1.91	232.00	236.28
16.16	6.76	3.54	2.23	1.59	235.00	314.40
17.09	7.10	3.60	2.39	1.51	158.00	162.98
10.55	4.62	2.17	1.89	1.15	315.00	251.60
10.12	6.49	3.54	2.19	1.62	90.90	103.21
6.03	4.29	1.81	1.82	0.99	170.00	256.87
6.00	7.05	3.58	2.47	1.45	195.40	325.36
4.61	4.30	2.00	1.60	1.25	195.00	244.01

4.61	4.30	2.00	1.60	1.25	195.00	218.08
14.56	4.84	2.04	2.33	0.88	114.00	108.76
14.56	4.84	2.04	2.33	0.88	114.00	258.78
17.70	3.70	2.22	0.85	2.61	110.00	225.39
4.61	5.01	3.45	1.15	3.00	219.00	221.79
18.38	5.41	2.88	1.97	1.46	191.00	161.91
4.61	5.30	3.50	1.10	3.18	94.00	98.48
19.36	3.98	1.11	2.01	0.55	131.00	238.02
10.12	3.78	1.86	1.60	1.16	200.00	192.25
19.36	3.98	1.11	2.01	0.55	131.00	192.61
14.51	6.20	4.10	1.50	2.73	123.00	213.00
17.79	3.39	1.17	1.78	0.66	127.50	196.55
7.50	5.50	2.60	2.40	1.08	151.00	181.53
0.15	10.85	5.33	3.49	1.53	193.20	107.27
19.08	4.49	2.36	1.43	1.65	119.90	246.31
4.61	6.04	3.85	1.64	2.35	129.00	235.11
4.61	4.97	2.86	1.28	2.23	229.00	191.43
8.93	9.10	6.76	1.80	3.76	274.00	178.29
14.42	4.30	2.30	1.50	1.53	204.00	168.97
8.45	6.07	3.30	1.69	1.95	170.00	186.19
8.11	5.39	2.48	2.25	1.10	280.10	150.21
17.08	5.45	2.82	1.81	1.56	159.00	152.16
14.28	3.39	1.42	1.48	0.96	73.00	185.79
9.73	5.77	2.66	2.45	1.09	167.00	162.93
6.40	6.25	3.43	2.15	1.60	203.00	192.89
16.21	5.83	3.48	1.80	1.93	170.00	159.23
14.67	3.70	2.29	1.04	2.20	215.00	141.47
12.38	4.60	2.11	2.01	1.05	132.00	217.71
11.09	4.00	2.30	1.30	1.77	150.00	184.88
16.45	3.21	1.49	1.16	1.28	21.90	211.98

16.10	5.57	4.10	0.99	4.14	126.00	199.21
13.17	5.22	3.17	1.71	1.85	213.00	155.16
17.06	6.00	2.70	2.60	1.04	165.00	140.47
17.04	6.14	2.73	2.66	1.03	255.40	208.03
8.23	6.45	3.90	1.93	2.02	151.00	129.69
12.92	5.38	2.73	1.92	1.42	124.90	188.86
14.54	7.17	3.94	2.31	1.71	195.30	209.18
10.65	6.78	3.80	2.43	1.56	261.00	166.38
9.08	4.14	2.29	1.57	1.46	147.00	195.63
15.63	8.91	5.41	2.44	2.22	163.10	307.94
10.40	6.36	3.66	2.17	1.69	141.00	157.97
6.21	7.33	4.29	2.36	1.82	100.40	229.07
6.21	1.29	0.86	0.25	3.44	21.00	175.31
20.25	8.78	4.66	2.99	1.56	183.10	70.22
6.21	5.43	2.62	2.00	1.31	86.70	203.34
13.86	5.21	3.29	1.29	2.55	94.00	214.28
9.21	3.22	1.30	1.61	0.81	50.40	79.88
6.21	20.06	18.07	1.62	11.15	148.00	153.26
15.62	5.06	2.39	1.88	1.27	145.60	188.56
9.60	5.37	2.42	2.11	1.15	133.60	136.13
12.52	5.69	2.40	2.53	0.95	163.40	117.91
11.84	4.87	2.28	1.64	1.39	108.70	147.74
14.75	23.02	21.29	0.68	31.31	89.50	139.08
15.81	8.21	6.21	1.43	4.34	170.00	232.22
NA	4.87	2.90	1.41	2.06	190.20	237.80
NA	4.93	2.91	1.47	1.98	234.50	210.03
NA	5.72	2.55	2.69	0.95	287.30	253.57
NA	5.29	3.21	1.63	1.97	355.10	232.22
NA	6.89	4.97	1.38	3.60	265.00	229.72
NA	7.42	4.20	2.07	2.03	217.20	313.48

NA	4.49	2.29	1.79	1.28	235.50	261.45
NA	7.98	4.41	2.89	1.53	313.50	157.71
NA	9.09	5.67	2.90	1.96	204.50	247.63
NA	6.61	2.86	3.31	0.86	247.60	239.53
NA	5.15	2.79	1.83	1.52	227.90	216.88
NA	6.25	3.51	2.41	1.46	295.90	209.15
NA	5.65	3.15	1.95	1.62	256.00	226.18
NA	5.34	2.25	2.71	0.83	151.40	201.25
NA	4.51	2.37	1.87	1.27	226.20	232.88
NA	4.78	2.81	1.60	1.76	333.90	230.18
NA	5.42	3.34	1.57	2.13	144.20	249.16
NA	7.16	4.92	1.37	3.59	220.70	238.45
NA	5.53	3.28	1.82	1.80	246.50	300.77
NA	8.61	6.34	1.82	3.48	220.30	240.45
NA	5.37	3.28	1.58	2.08	156.50	207.90
NA	7.33	4.44	2.25	1.97	203.30	246.54
NA	5.84	3.47	1.99	1.74	250.50	204.38
NA	12.71	10.67	1.43	7.46	278.90	244.72
NA	4.71	2.75	1.55	1.77	197.10	264.52
NA	7.89	4.96	2.29	2.17	257.00	258.47
NA	6.84	4.84	1.37	3.53	267.20	281.30
NA	6.22	3.28	2.33	1.41	210.90	174.05
NA	4.67	2.86	1.40	2.04	220.30	198.99
NA	5.11	3.01	1.72	1.75	300.70	224.41
NA	6.64	3.95	2.22	1.78	228.10	178.14
NA	7.65	3.81	3.09	1.23	244.00	185.42
NA	8.00	5.77	1.65	3.50	277.70	173.41
NA	6.50	4.41	1.67	2.64	156.60	144.80
NA	6.80	3.85	2.13	1.81	282.00	171.20
NA	5.24	2.90	1.71	1.70	190.70	147.11

NA	8.76	5.35	2.64	2.03	281.60	168.81
NA	8.53	4.96	2.95	1.68	219.80	151.12
NA	5.08	2.80	1.86	1.51	220.30	208.82
NA	6.12	3.90	1.60	2.44	153.70	144.57
NA	4.89	2.97	1.54	1.93	322.50	168.14
NA	7.96	4.18	3.09	1.35	288.50	175.12
NA	5.81	4.32	1.07	4.04	204.30	157.87
NA	4.51	2.76	1.46	1.89	248.60	171.60
